# Mucin1 as a potential molecule for cancer immunotherapy and targeted therapy

**DOI:** 10.7150/jca.88261

**Published:** 2024-01-01

**Authors:** Xiaohan Tong, Chunyan Dong, Shujing Liang

**Affiliations:** Department of Oncology, Shanghai East Hospital, School of Medicine, Tongji University, Shanghai 200092, China.

**Keywords:** MUC1, cancer, immunotherapy, targeted therapy, combination therapy

## Abstract

Mucin1 is a highly glycosylated type 1 transmembrane mucin that ranks second among 75 tumor-related antigens published by the National Cancer Institute, and has been identified as a possible therapeutic target over the past 30 years. MUC1 plays an important role in malignant transformation and disease evolution, including cell proliferation, survival, self-renewal, and metastatic invasion. MUC1 has been shown to interact with diverse effectors such as β-catenin, receptor tyrosine kinases, and cellular-abelsongene, which are of importance in the pathogenesis of various malignant tumors. Targeting MUC1 has been shown to be an effective way to induce tumor cell death in vivo and in vitro models. In recent years, a number of therapeutic strategies targeting MUC1 have been developed and their value for tumor therapy have been demonstrated experimentally. This review summarizes recent findings on the structure of MUC1, its expression in different tumors and its involved mechanism pathways, with emphasis on new progress in cancer therapy which related MUC1 in the past decade and evaluates their therapeutic effect.

## Background

The mucin family to which Mucin1 (MUC1) belongs is a large family of highly glycosylated proteins^1^. In healthy tissues, MUC1 covers the surface of all epithelial cells to create a physical barrier which could protect the cells from extreme environmental conditions and prevent pathogenic access^2^. It is also involved in lubrication^3^, cell surface hydration^4^, and protection from degradative enzymes^5^. These functions mainly depend on its extracellular domain, which has a series of 20 amino acid repeat units that are highly glycosylated in normal cells^6^ and abnormally glycosylated^7^ or lost in polarization^8^ in cancer cells.

In addition, the intracellular domain of MUC1 is involved in multiple pathways related to tumor occurrence^9^, progression and metastasis^10^, ^11^, and is related to subsequent treatments such as the mechanism of drug resistance of chemotherapy drugs^12^. Notably, MUC1 is overexpressed in a variety of adenocarcinomas, including breast, ovary, colon, rectum and prostate^13^-^15^. Therefore, the study of MUC1 and the preparation of anti-tumor drugs against MUC1 are of great importance for the comprehensive treatment of cancer. Fortunately, a large number of studies have been carried out in the past, including the preparation of anti-MUC1 monoclonal antibody and its application in chimeric antibody receptor T cell (CAR T) therapy, vaccine using MUC1 as immunogen, and combination therapy. Here, we will describe and comment on them one by one.

## The Structure of MUC1

### MUC1 structure in normal cells

MUC1 consists of two subunits: the longer N-terminal subunit (MUC1-N) and the shorter C-terminal subunit (MUC1-C).

The N-terminal subunit is located on the membrane surface and contains the signal peptide, the (30-90) variable number tandem repeat (VNTR) region, and the sea urchin sperm protein, enterokinase, and agrin (SEA) domain. Each VNTR region consists of 12-125 repeats of a 20 amino acid sequence, which has five possible O-glycosylation sites per repeat, and the VNTR region is rich in serine(Ser) and threonine(Thr) residues, which are the sites of O-glycosylation^16^, ^17^. The VNTR region is flanked by imperfect repeat (IR) sequences, which are short degenerate sequences with similarity to the VNTR region, also contain Ser/Thr residues^18^; and contain few asparagine residues, the sites of N-linked glycosylation^13^, ^19^-^21^. Therefore, glycosylation exerts a vital influence on the preferred conformation of VTNR peptides, which has been described to tumor-associated antigens structures.

MUC1-C consists of 58 amino acids of the external domain (SEA), the transmembrane domain and the intrinsically disordered cytoplasmic domain (MUC1-CD)^22^. MUC1-CD includes a CQC motif, which is essential for the formation of MUC1-C homodimers and their import into the nucleus. The short and highly conserved cytoplasmic domain contains seven tyrosine residues and several Ser and Thr residues, which represent potential docking sites for proteins with Src homology two domains and recognition sites for receptor tyrosine and other kinases. This includes epidermal growth factor receptor (ErbB) receptors such as protein kinase C delta, glycogen synthase kinase 3β (GSK3β), and epidermal growth factor receptor (EGFR)^23^.

### Tumor-associated MUC1

**Figure 1:**
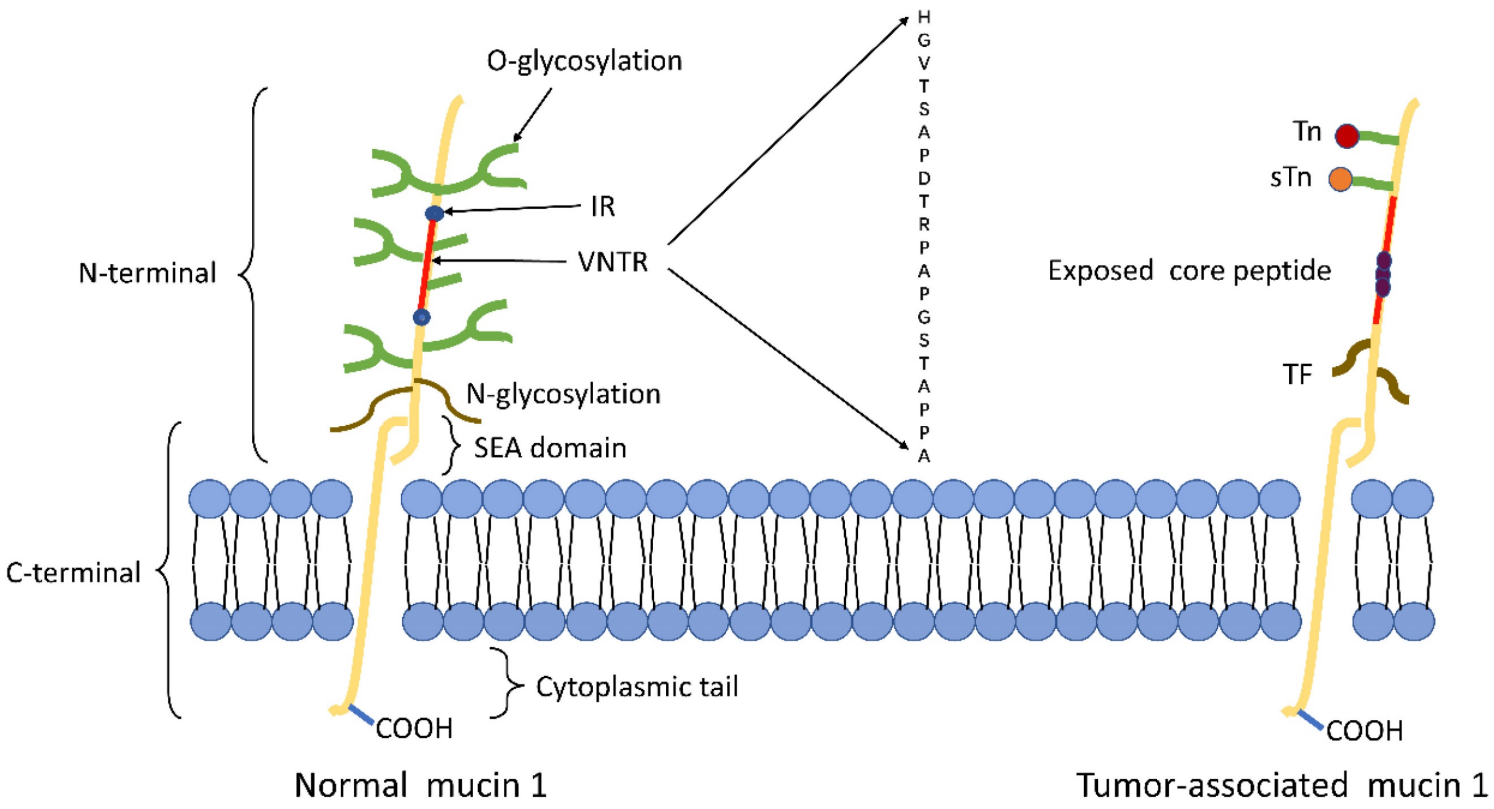
Schematic representation of MUC1 structure. (A) The structure of MUC1 in normal tissues. MUC1-N contains the signal peptide, VNTR region (red), and SEA domain. The VNTR region of MUC1-N is composed of 20 amino acids that are extensively O-glycosylated (green) at the serine and threonine residues. The VNTR region is flanked by IR (blue) sequences. MUC1-N and MUC1-C are sparingly N-glycosylated (brown) at asparagine residues. MUC1-C consists of the SEA, the transmembrane domain and the MUC1-CD. (B) The structure of MUC1 in tumor tissues. The abnormal glycosylation of the tumor-associated MUC1 leads to the formation of new carbohydrate side chains [Tn (red), sTn (orange), TF (black)] and the exposure of core peptide(black).

The structure of tumor-associated MUC1 was significantly altered compared to that of normal cell surface MUC1. First of all, MUC1 is overexpressed in a number of different cancers^24^, ^25^. The expression level of MUC1 on the tumor cell surface is 10-40 times higher than the normal cell surface^26^, and this overexpression is often associated with poor tumor prognosis. Secondly, the loss of cell polarity causes the expression of MUC1 to be discovered over the cell surface and within the cytoplasm^8^. Third, reduced glycosylation. In normal cells, MUC1 is highly glycosylated and peptide core are masked by the sugar moieties, while the extension of cancer-associated MUC1 carbohydrate side chains terminates prematurely or forms truncated sugar branches, which leads to exposure of new epitopes in core protein^27^, ^28^. Finally, incomplete carbohydrate of the carbohydrate side chains lead to the formation of new carbohydrate side chains [Thomsen-Friedenreich (Tf or T), Thomsen nouvelle (Tn), and sialyl-Tn (STN)]^29^ (Fig.1).

## The function of the MUC1

### MUC1 in normal tissues

MUC1 has a widespread tissue distribution whose primary functions are to hydrate, protect, and lubricate the epithelial surfaces of the ducts within the human body^30^. In the epithelial lining of the stomach, MUC1 can resist microorganisms like Helicobacter pylori^31^; Bacterial adhesins can also bind MUC1 on the cell surface, a process that usually prevents infection^32^. Furthermore, the protective effect of MUC1 is important to defend oral epithelial surfaces from various noxious, pathogenic and non-pathogenic microbes^33^. In the other hand, ocular surface epithelial cells produce and secrete MUC1 that form a hydrophilic barrier for protection and lubrication of the eye^34^.

### MUC1 in tumor tissues

MUC1 is associated with a variety of tumor transformation-related and progression-related signaling pathways, and this function mainly depends on its MUC1-C domain (Table 1).

#### MUC1 in breast cancer

In breast cancer, with the loss of polarity associated with epithelial stress response or transformation, MUC1-C and EGFR are expressed over the entire cell membrane and are repositioned to form complexes. The interaction between MUC1 and EGFR at the cell membrane has been shown to increase EGFR internalization and recycling^35^. MUC1-C also promotes EGFR-dependent activation of the Phosphoinositide 3-kinase (PI3K)→Protein kinase B (AKT) pathway, which functions in the activation of diverse effectors that promote growth and survival^36^. Therefore, MUC1 contributes to EGFR-mediated tumorigenesis. MUC1 and vascular endothelial growth factor (VEGF) expression in human breast cancer are highly correlated, promoting the synthesis and secretion of VEGF through the AKT signaling pathway^37^.

MUC1-C associates with STAT1 in cells through direct binding of the MUC1-C cytoplasmic domain to the STAT1 DNA-binding domain. The MUC1-C/STAT1 interaction promotes the activation of STAT1 target genes, including *MUC1* itself^38^. Like STAT1, MUC1-C interacts with STAT3 in the similar way^39^. Studies in breast cancer cells have shown that MUC1 associates with ErbB2 and that this interaction is increased by heregulin stimulation^17^, ^40^. In fact, silence of MUC1 down-regulates ErbB2 activation and reverses trastuzumab resistance^41^.

MUC1-C activates the nuclear factor kB p65(NF-kB p65) pathway in the pro-inflammatory factor Transforming growth factor-b-activated kinase-1 (TAK1) → IkB kinase (IKK) cancer cells or directly binds to NF-κB p65 to drive its downstream target genes^42^, inducing the epithelial-mesenchymal transformation (EMT) in basal B cells^43^. Besides, MUC1-C also enhances the transcription rate of the immune checkpoint ligand programmed cell death-Ligand 1 (PD-L1) in triple-negative breast cancer (TNBC) cells by recruiting MYC and NF-kB p65.

β -catenin binds directly to the SxxSSL site in the MUC1-C cytoplasmic domain, which is the first link between MUC1 and the Wingless Integrated (Wnt) pathway. MUC1-C was also shown to destabilize p53 and increase stability and activity of β-catenin and Estrogen receptors alpha (Erα); thereby aberrant activation of Wnt target genes, such as Cyclin D1 (*CCND1*) and *MYC*, *CCND1* plays an important role in breast neogenesis^44^. In turn, activation of the Wnt pathway inhibits GSK3β and thereby results in the stabilization of β-catenin.

#### MUC1 in hematological malignancies

MUC1 could induce myeloid suppressor cells (MDSCs) in acute myeloid/myelogenous leukemia (AML) cells^45^. MDSCs, which derive from immature monocytes and suppress effector T cells, were induced by extracellular vesicles from AML cells, MUC1 is a critical component of these vesicles^13^, ^46^. The MUC1-C interacts with receptor tyrosine kinases (RTKs), at the cell membrane resulting in phosphorylation of fms-like tyrosine kinase 3 (FLT3), and subsequent activation of downstream effectors, such as AKT, extracellular regulated protein kinases (ERK) and STAT5 signaling. FLT3 functions as an important oncogene in AML and is associated with poor outcomes following standard chemotherapy^44^. In chronic myelocytic leukemia (CML), inhibition of the MUC1-C oncoprotein disrupts redox balance and thereby 1) decrease break-point cluster region protein (BCR)/Tyrosine-protein kinase (ABL) levels, 2) downregulate β-catenin expression, and 3) induce terminal myeloid cell differentiation, thus avoid CML to enter the acute phase^47^.

In multiple myeloma, inhibition of MUC1-C is associated with increases in reactive oxygen species (ROS) and significant down-regulation of the TP53-induced glycolysis and apoptosis regulator (TIGAR). The present results further show that MUC1-C inhibition in multiple myeloma cells is associated with depletion of Nicotinamide Adenine Dinucleotide Phosphate (NADPH) and glutathione (GSH), which in turn promotes the induction of late apoptosis/necrosis in the response to oxidative stress^15^.

#### MUC1 in pancreatic cancer

In pancreatic cancer, MUC1-C could target N-Acetylgalactosamine(GalNAc)-T5, a glycosyltransferase associated with tumor suppression in pancreatic cancer, to exert carcinogenic activity^48^. Galectin-3 has been shown to regulate MUC1 and EGFR internalization and subcellular localization, ERK1,2 activation down-stream of the EGFR and EGFR nuclear translocation in pancreatic cancer cells^49^, ^50^. MUC1 induces angiogenesis in tumor microenvironments by increasing the expression of neuropilin-1(NRP1, a co-receptor of VEGF) and its ligand VEGF^51^. Silencing the expression of MUC1 can also inhibit the migration and invasion of PANC-1 cells and induce apoptosis by down-regulating the transcription factor Slug^52^.

MUC1 regulates the hypoxia response of pancreatic cancer cells and reduces the sensitivity of pancreatic cancer cells to gemcitabine by regulating the expression of hypoxia-inducible factor-1 α (HIF-1α) 53. In addition, MUC1 binds to HIF-1α and P300 in a hypoxic-dependent manner and acts as a metabolic regulator to help tumor cells survive and proliferate under such conditions^54^.

MUC1-mediated nucleotide metabolism also plays a key role in promoting radiotherapy resistance in pancreatic cancer and can inhibit effective targeting through glycolysis^55^, ^56^. In a human pancreatic cancer cell model, the cytoplasmic caudal motif of *MUC1* directly binds to the promoter region of multidrug resistance (*MDR*) gene ATP-binding cassette (*ABC-C1*) gene and upregulates the level of multidrug resistance-associated protein 1 (MRP1) protein encoded by Abcc1 through the Akt-dependent pathway, MRP1-9 is a class of the ABC transporters that have been positively linked to the MDR phenotype in cancer cells. As a result, MUC1 is directly related to chemotherapy resistance of pancreatic cancer cells^57^.

#### MUC1 in lung cancer

Non-small cell lung cancers (NSCLC) are associated with constitutive activation of the PI3K-Akt-mTOR pathway. The direct binding of the MUC1-C cytoplasmic domain to the PI3K P85 SH2 domain leads to a conformational change of P85, thereby activating the PI3K P110 catalytic subunit, which is related to the activation of the PI3K->Akt pathway 35. In addition, MUC1-C is also a prerequisite for the expression of PD-L1 in NSCLC cells. Audrey Bouillez et al. show that MUC1-C increases NF-κB p65 occupancy on the *CD274/PD-L1* promoter and thereby drives *CD274* transcription, supporting the *MUC1-C NF-κB P65* pathway to drive *PD-L1* expression. This pathway also induces toll-like receptor 7 (*TLR7*) expression in NSCLC cells. Moreover, MUC1-C-induced activation of NF-κB→ZEB1 signaling represses the toll-like receptor 9 (*TLR9*), interferon gamma (*IFNG*), Monocyte chemoattractant protein-1 (*MCP-1*) and granulocyte-macrophage colony-stimulating factor (*GM-CSF*) genes, and that this signature is associated with decreases in overall survival^58^.

#### MUC1 in other cancers

In hepatocellular carcinoma (HCC) cells, MUC1 can promote radio resistance by activating the JAK2/STAT3 signaling pathway^59^. In colon cancer, Siglec-9 expressed on immune cells may facilitate tumor progression through ligation with MUC1^60^. MUC1 also interacts with immune cells, which act as a novel T cell costimulatory molecule involved in immune regulation through binding to activator protein-1 (AP-1) transcription factors c-Fos and c-Jun^61^.

## MUC1 and treatment

### Immunotherapy

MUC1 has been associated with immune checkpoint genes^21^, neoantigens, and certain prognostic indicators of immunotherapy such as tumor mutational burden (TMB) and microsatellite instability (MSI); MUC1 is itself a T-cell costimulatory molecule^61^, suggesting that MUC1 may also be a target of immunotherapy.

#### Antibodies to glycopeptide epitopes in the TR domain

Many MUC1-specific antibodies react with epitopes within the PDTRP sequence and reactivity is affected by glycosylation. The PankoMab antibody reacts with a conformational epitope where the threonine in PDTRP carries the Tn or T glycan and selectively reacts with the cancer mucin. PankoMab-GEX has been humanized and glyco-optimized for improved antibody-dependent cell-mediated cytotoxicity (ADCC) and antibody-dependent cellular phagocytosis (ADCP) activity and enhanced NK cell killing^13^. In a Phase I trial (NCT01222624), 74 patients with advanced MUC1-positive carcinomas received PankoMab-GEX intravenously until disease progression. PankoMab-GEX showed promising anti-tumor activity in advanced disease, especially in ovarian and NSCLC^62^. The HMFG-1 monoclonal antibody was raised against human milk fat globules, a rich source of MUC1 and was also shown to bind the exposed PDTR sequence^63^. The humanized version of this antibody named HuHMFG-1, which well tolerance was demonstrated successfully in a phase I clinical trial of breast cancer (NCT00096057)^64^ but poor clinical efficacy was shown in a phase II clinical trial of breast cancer (NCT00770354)^65^.

While MUC1-N can be shed from the surface of tumor cells, and the plasma level of MUC1-N is increased in patients with metastatic cancer. So, the extracellular pool of MUC1-N represents a barrier for the delivery of a monoclonal antibody (Mab) to tumor cell surfaces to induce ADCC. Therefore, the MUC1-N circulating pool must be overcome in order to target the surface of tumor cells expressing MUC1.

#### MUC1 CARs

Little is known regarding the specifics of tumor-associated MUC1 (tMUC1) epitope, thus far, tumor MUC1 targeting is mostly restricted to the VNTR region. A novel monoclonal antibody TAB004 was developed, which is highly specific for the tMUC1 and does not recognize normal epithelia^18^, ^66^. The second-generation MUC28z CAR T cells derived from scFv of TAB004 significantly lysed TNBC tumor cells despite with high surface expression of programmed death 1 (PD1), and this activity strongly correlated with level of surface expression of tMUC1^18^.

More recently, a CAR fusion protein with a humanized version of the 5E5 antibody has been developed. 5E5 reacts preferentially with the GSTA sequence carrying Tn and to a lesser extent STn 20. Notably, MUC1 is also expressed in normal cells, and the MUC1 protein core and salivary acidification epitopes are identical in cancer cells and T cells, except that the peptide exposed to cancer cells due to abnormal glycosylation was not exposed to activated T cells. While targeting MUC1 on T cells can lead to severe "non-tumor" recognition, known as "targeted non-tumor" toxicity, leading to severe normal organ damage and even death^66^, ^67^. The specificity of the 5E5 CAR, which does not bind the glycoform of MUC1 on T cells, could circumvent problems that might be encountered with other MUC1 CARs^13^, ^20^, ^68^.

The use of dual antigen-specific T cells improves the "targeted non-tumor" toxicity of CAR immunotherapy. Erbb2-specific Cars were constructed using single-chain antibodies against ErbB2 Mab and the CD3ζ signaling domain. The MUC1-specific CAR conjugated to the CD28 domain against MUC1 single-chain antibodies. Combining the two CARs using a single SFG vector containing an intermediate T2A sequence produced a CAR with dual targeting of ErbB2 and MUC1, which was named ITH. ITH can deliver complementary signals, leading to target-dependent cytotoxicity and co-enhanced proliferation, but with limited IL-2 production, which may be due to conformational changes in single-chain antibodies. Future studies may attempt to address this problem by improving the design of CARs^69^, ^70^. Xinru Wei also demonstrated enhanced efficacy of a combination of both prostate stem cell antigen (PSCA)- and MUC1-targeted CAR T cells against double-positive NSCLC samples^71^. Finally, dual-targeted CAR approaches do enhance the anticancer efficacy of CAR T cells while minimizing extra tumoral side effects in normal tissues that carry a single antigen.

#### MUC1-Bi bispecific antibodies

Bispecific antibodies combine specificities of two antibodies and simultaneously target two different antigens or epitopes. A new bispecific antibody MUC1-Bi-2 was constructed by linking a single domain antibody, anti-MUC1-VHH and anti-CD16-VHH. In vitro, the Muc1-Bi bispecific antibodies can recruit natural killer (NK) cells to drive potent and specific cell killing of Muc1-overexpressing tumor cells. In xenograft model, the Muc1-Bi bispecific antibodies can suppress tumor growth in the presence of human peripheral blood mononuclear cells (PBMC)^72^.

#### MUC1-based cancer vaccines

MUC1-based cancer vaccines include subunit vaccines, DNA vaccines, viral vector vaccines, DC vaccines and glycopeptide vaccines (Table 2).

##### Subunit vaccines

The exposed epitope APDTRP in aberrantly glycosylated MUC1 can be recognized by multiple anti-MUC1 antibodies and then activate tumor antigen-specific CTLs. Therefore, MUC1 VNTR peptide epitopes can be used in subunit vaccines, which induce adaptive immune responses. These vaccines either conjugate or mix with a variety of immune agonists, including keyhole limpet hemocyanin (KLH)^73^, mannan^74^, ^75^, bacillus Calmette-Guerin (BCG)^76^, maltose binding protein (MBP)^77^, ^78^, Quillaja saponaria extract (QS-21), or SB-AS2^79^, in order to improve the immunogenicity of vaccination. These strategies are not only safe, but also enhance the effectiveness of vaccines^80^.

Tecemotide (L-BLP25), a 25-aa VNTR MUC1 peptide in a liposomal formulation with a TLR-4 agonist, monophosphorylate lipid A. The results support the ability of L-BLP25 immunotherapy to elicit MUC1-specific responses in a substantial proportion of people with MM, generating a combined and diversified T- and B-cell immune response^81^, ^82^. In phase II trials based on breast cancer and non-small cell lung cancer, L-BLP25 has shown safety and potential clinical efficacy^83^, ^84^. On the basis of these promising findings, the researchers initiated the START (Stimulating Targeted Antigenic Response To NSCLC) study to assess the efficacy of L-BLP25 when compared with placebo as a maintenance therapy in patients with stage III NSCLC who have received chemoradiotherapy. Results showed no significant difference in overall survival after chemoradiotherapy with L-BLP25 compared with placebo, but the data suggest that the subgroup of patients who received initial concurrent chemoradiotherapy might benefit from maintenance L-BLP25, which warrants further study^85^.

In patients without cancer but with a history of premalignant lesions (advanced colonic adenomas, precursors to colon cancer), Prophylactic administration of MUC1 peptide vaccine showed high levels of anti-MUC1 immunoglobulin G (IgG) and long-lasting immune memory without toxicity. Lack of response was correlated with high levels of circulating MDSCs prevaccination. This shows promise as an alternative prevention strategy for patients at high risk of cancer with MUC1 subunit adjuvant vaccines^86^.

Although subunit vaccines have the advantage of being well tolerated, they are difficult to induce predictable or consistent adaptive immune responses, this may be related to low immunogenicity of selected antigens, advanced disease of trial participants, and simple vaccine regiments.

##### RNA vaccines

RNA-based vaccines have attracted much attention recently, certain advantages over other vaccines may be offered. RNA vaccines can induce stronger immune response^87^, safer than DNA vaccines, and can be rapidly and massively produced, which is the biggest advantage of RNA vaccines^88^. Nanoparticles (NPs) could deliver MUC1-encoding mRNA to DCs in lymph nodes, as suggested by in vivo research, the NP-based mRNA vaccine induce a potent antigen-specific T-lymphocyte response with in vivo cytotoxicity for the resistance of breast cancer cells^89^.

##### DNA vaccines

Many experiments have demonstrated the safety and efficacy of MUC1 DNA vaccine^90^-^92^, but poor immunogenicity limits its application. A DNA vaccine consisting of MUC1 and surviving was successfully constructed, and further investigation confirmed that the combined DNA vaccine elicits enhanced CTL activity and results in better tumor growth inhibition efficiency compared with single antigen vaccine^93^, ^94^. Previous studies have improved vaccines by adding DC maturation signals to the MUC1 cDNA. one approach consisted of coupling MUC1 DNA with DNA corresponding to the expression of a heat shock protein70 (HSP70), MUC1/HSP70-coupled DNA increased the capacity of DC to induce effective cytotoxic T-cell responses and inhibited tumor cell growth^95^.

##### Viral vector vaccines

The major difference between a viral vector vaccine and DNA vaccine is that they are different carriers, but they have the similar role mechanisms in eliciting the immune responses^96^. TG4010 is a modified Vaccinia Ankara strain expressing a full-length MUC1 (containing five TRs) and interleukin-2. therefore, Class I epitopes outside of the TR domain can be presented^97^, ^98^. TG4010 is being used against several types of cancer, including prostate cancer^99^, renal cell carcinoma (RCC)^100^, and NSCLC. The best clinical responses using TG4010 were observed for lung cancer, two phase II clinical trials suggested that TG4010 plus chemotherapy was a worthy exploration option for the treatment of advanced NSCLC^101^, ^102^. Recently, a novel vaccine using adenovirus 5(Ad5) vectors [E1-,E2b-] to target three TAAs—prostate specific antigen (PSA), brachyury and MUC-1—has been developed for the first time in human trials in patients with metastatic castration-resistant prostate cancer, which was well tolerated and demonstrated clinical activity^103^.

##### Dendritic cell (DC) vaccines

Since the immunogenicity of vaccines is highly dependent on uptake by DCs, developing DCs as antitumor vaccines is a powerful strategy for cancer prevention and immunotherapy^96^. A number of phase I and Phase II clinical trials have been conducted to investigate the safety and efficacy of DC vaccines with different strategies in a variety of tumors, such as resected metastases of colorectal cancer^104^, Ovarian Cancer^105^,non-small cell lung cancer^106^ and pancreatic ductal adenocarcinoma^107^ et al. Cvac (autologous dendritic cells (AuDCs) incubated with mannosylated MUC1 protein (M-FP)) is well tolerated and active in epithelial ovarian cancer^108^. Recently, DC-based Tn-MUC1 glycopeptide vaccine has shown safety in patients with non-metastatic castration resistant prostate cancer, eleven of the 16 patients had a significant increase in mean prostate specific antigen doubling time (PSADT) compared to pre-vaccination, indicating the biological activity of the vaccine. But none of the subjects showed a decrease in PSA levels, in fact, PSA increased in all patients^94^. So, the strategies for DC vaccine preparation need to be optimized.

In preclinical studies, fusion cells have been shown to more potently stimulate antitumor immunity than single antigen peptide vaccines, and have showed the ability to eradicate established metastatic disease. Trial showed that a fusion cell vaccine consisting of patient-derived myeloma cells and AuDCs is safe in multiple myeloma patients treated with autologous stem cell transplantation, also showed the vaccine mediated T cell amplification of myeloma antigen related with a specific evidence of observed (MUC1) + T cells increased 15 times^109^.

Although DC vaccines have shown considerable potential in some malignant tumors in clinical trials, unfortunately, these vaccines still have not entered phase III trials and issues such as induction and regulation of DC maturation, dosage and immune route may need to be addressed^96^, ^110^. At present, with a deeper understanding of the reconstituted tolerance of vaccines caused by immune checkpoints, future work needs to combine vaccination with blocking PD-L1 / PD-1 pathway to evaluate the efficacy.

##### Glycopeptide vaccines

Aberrant glycopeptides in the tandem repeat region are particularly attractive target structures, such as T, Tn and STn. Since the binding affinity of natural glycopeptides is not sufficient to stimulate the proliferation and differentiation of primitive B cells into plasma cells that secrete antibodies--which leads to the immune system's self-tolerance, synthetic glycopeptide vaccines are the best option. MUC1 glycopeptide has been widely bound to a variety of immune stimulants, with or without adjuvants. Potential immune stimulants such as carrier protein^111^, T cell epitopes peptide^112^-^116^, Toll-like receptor 2 lipopeptide ligands^117^. Tetanus toxoid (TTox) has a variety of T cell epitopes and is most widely used in the preparation of synthetic glycopeptide vaccines. Three Tn sugar antigens linked to glycosylation sites bind tetanus toxoid to produce a vaccine which, without support by an adjuvant, elicited the strongest immune response (endpoint titer 100 000) and antibodies binding to tumor cells. Thus, it is considered a most promising lead for the development of fully synthetic antitumor vaccines^113^.

Despite the advances achieved in recent years by using these multicomponent vaccines in the development of stronger immune response, the weakness of easy tolerance of therapeutic vaccines remains a barrier. An attractive approach to tackle this issue may be the utilization of unnatural derivatives. In principle, these derivatives will be more resistant to enzymatic degradation, which could be translated into stronger and longer-lasting immunogenicity and protective efficacy^118^, ^119^.

### Targeted therapy

#### MUC1-C inhibitor

The MUC1-C cytoplasmic domain contains a CQC motif that is necessary for its homodimerization and subsequent nuclear localization. Based on these findings, a cell-penetrating peptide drug was developed to inhibit MUC1-C homodimerization and its oncogenic function^44^. The MUC1-C inhibitor GO-203 contains the endogenous MUC1-C CQCRRKN amino acid sequence linked to nine arginine residues at the N-terminus for cell permeability. GO-203 could block the interaction between MUC1-C and its downstream targets in breast and lung cancer cells, inhibiting cell self-renewal^120^.

#### Targeted MUC1-C

**Figure 2:**
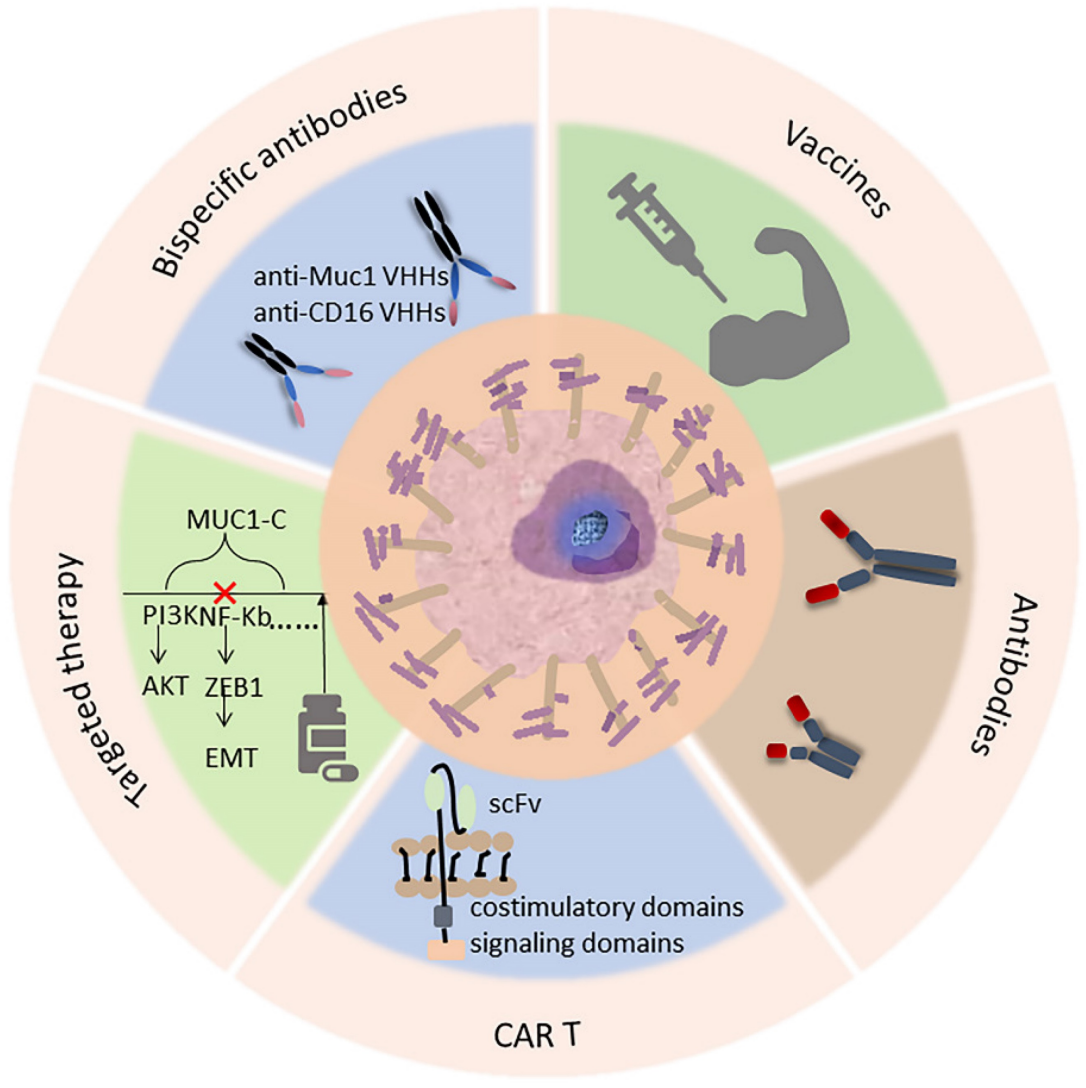
Current and potential clinical approaches targeting MUC1 in cancer, including immunotherapy (antibody, CAR-T therapy, vaccine) as well as targeted therapy, an example of treatment is listed in each box below.

In addition, as an attractive target, monoclonal antibot-based therapies are being developed for MUC1-C. For example, MAb 3D1 is an antibody against the non-shed MUC1 C-terminal subunit. It binds to monomethyl whey protein E (MMAE) to form mAb 3D1-MMAE antibody-drug conjugate (ADC), which kills MUC1-C positive cells in vitro while being non-toxic to MUC1 transgenic (MUC1.Tg) mice and active against human HCC827 lung tumors^121^. There is a monoclonal antibody specific for the extracellular region of the MUC1 subunit MUC1-C (anti-hMUC1), which recognizes the MUC1-C protein in pancreatic cancer cells, thereby inhibiting epidermal growth factor (EGF)-mediated ERK phosphorylation and CCND1 expression and inhibiting the growth of pancreatic tumor in mice^122^. The use of anti-hMUC1 antibody in breast cancer also significantly inhibits the proliferation of breast cancer cells^123^. SAR566658 ADC is a cytotoxic maytansinoid derivative drug, DM4 conjugated through a cleavable linker to the humanized DS6 (HuDS6), which can recognize MUC1's CA6 sialoglycotope. SAR566658 showed in vivo antitumor efficacy against CA6-positive human pancreas, cervix, bladder, and ovary tumor xenografts and against three breast patient-derived xenografts^124^. So, this is a promising targeted therapy that could be further developed (Fig. 2).

### Nanomedicine

The more accurate and efficient choices can be provided by nanomedicine technology compared with traditional treatments. MUC1 is known as one of the important tumor markers, so nanocarriers-based smart drug delivery systems are often used to deliver anti-MUC1 preparations for tumor therapy.

The MUC1 aptamers have been successfully used in NPs formulation, poly(lactic-co-glycolic acid) NPs loaded with the anti-MUC1 aptamer and labeled with technetium-99m showed good imaging properties in induced animal models with TNBC^125^. Furthermore, MUC1 aptamer-Cytochrome C aptamer-Graphene oxide could effectively induce cell programmed death in MDA-MB-231 and MCF-7 cells^126^. Recent studies have demonstrated that integrated aptamers as functional units could selectively target transmembrane glycoprotein MUC1, which was highly expressed in tumor cell membrane, thus enhancing the ability of nano-drugs delivery^127^-^129^.

The polyethylene glycol-polycaprolactone (PEG-PCL) polymer loaded with MUC1 inhibitor and DOX exhibited reliable safety and excellent anti-tumor growth ability in ascites carcinoma murine model^130^. Signal regulatory protein α siRNA and pMUC1 (a therapeutic pDNA vaccine) encapsulated with trimethyl chitosan could accumulated in the macrophages in lymph nodes and tumor tissues via oral administration, resulting in a strong cellular immune response^131^.

### Combination therapy

Compared with monotherapy, combination therapy has a more significant tumor inhibition effect. Currently, the main combination therapy focuses on vaccine combined with other drugs to enhance the anti-tumor effect.

Studies have combined a vaccine consisting of the MUC1 core peptide with indomethacin to stimulate tumor-specific immune responses and alleviate the immunosuppressive microenvironment within breast tumors. Indomethacin did not increase tumor-specific immune responses compared to vaccine alone, but the combination of vaccine and drug reduced tumor cell proliferation and increased tumor cell apoptosis, making the cells susceptible to killing by immune cells^132^.

Radiotherapy is an extremely important option of tumor treatments, so combined radiotherapy is also the focus of current researches. Anti-MUC1-C/NPs combined with radiation therapy could enhance the effectiveness of radiation therapy in breast cancer^133^. Local radiotherapy combined with CV9202 which is an active cancer immunotherapeutic containing sequence-optimized mRNAs encoding MUC-1 antigen could synergistically treat NSCLC^134^. It has also been demonstrated that TG4010 can be combined with radiotherapy, and radiation enhances the efficacy of TG4010, and vaccination before radiation is more effective than reverse vaccination^97^. In addition, the viral vector vaccine TG4010, significantly improves progress-free survival in patients with advanced NSCLC when used in combination with chemotherapy^102^. At the same time, with the development of immune checkpoint inhibitors, future studies will also focus on whether the activity of TG4010 is affected by PD-L1 expression levels in tumor specimens, and TG4010 will also be administered in combination with immune checkpoint blockers to test efficacy.

Studies have shown that tumor suppression is significantly more effective when LCP vaccine with MUC1 mRNA loaded is combined with antibody blocking of CTLA-4. Blocking CTLA-4 leads to Akt phosphorylation, which may promote effective T-cell activation and ultimately improve therapeutic efficacy when combined with the vaccine^89^. Blocking of various cancer vaccine binding inhibitory pathways has been validated in preclinical models and has been shown to enhance T cell infiltration in a variety of tumors^135^.

## Conclusion

MUC1 is a tumor-associated antigen worthy of study. It is involved in a variety of pathways in tumor genesis and development, and its function mainly depends on MUC1-C. The glycopeptide epitopes on the surface of MUC1-N can also be used to prepare monoclonal antibodies. Due to its easy shedding from the surface of tumor cells, the therapeutic effect of antibodies is affected. Therefore, the current research focus is still MUC1-C, and many monoclonal antibodies and inhibitors have been developed for MUC1-C. In addition, many advances have been made in MUC1 CARs. These experimental immunotherapies targeting MUC1 are designed to induce anti-MUC1 antibodies to clear tumor cells. Nevertheless, their specific mechanisms in immune regulation have not been conceived. There is a clear need to understand the potential impact of the production of anti-MUC1 antibodies on the T-cell response in cancer patients to modulate the T-cell response in a variety of tumors.

At present, the vaccine against MUC1 has also achieved many results, a large number of preclinical studies and clinical trials have proved the safety and clinical activity of vaccines based on MUC1, and could induce T cell reactions. A number of strategies have been developed to address the problem of weak vaccine efficacy, such as adding adjuvants and immune stimulants to the vaccine platform, optimizing viral vectors, selecting nanomaterials to modify the vaccine, and targeting multiple antigens et.al. All these strategies have been proved effective in experiments; Nano delivery systems loaded with MUC1 preparations can not only improve drug delivery efficiency, but also loaded with multiple drugs to achieve synergistic treatment. The focus of current researches on using MIUC1 aptamers to modify nanomedicines. Combination therapy can significantly enhance the therapeutic effect, in particularly, when combined with emerging nonspecific immunotherapy approaches, such as PD-1 pathway inhibitors, showing promise. And the combination therapy was more effective than monotherapy in prolonging the survival of patients with advanced disease. But we still have a long way to go in terms of integrating anti-MUC1 into routine therapy.

## Figures and Tables

**Table 1:** Different molecular properties and function of MUC1 in health or cancer tissues.

	Normal MUC1	Tumor associated MUC1
Glycosylation	High glycosylation	Hypoglycosylation
Localization	Apical surface of normal epithelial cells	Cell surface, cytoplasm, nuclei and mitochondria of cancer cells
Expression	Normal expression	Overexpressed in malignancy
Functions	Barrier	Pro- or anti-inflammatory role
	Lubrication	Chemoresistance and resistance to apoptosis
	Cell surface hydration	Cancer cell invasion and metastasis effect
	Protection of cells from degrading enzymes	Stimulate angiogenesis

**Table 2:** Phase III and Phase II MUC1-targeted clinical trials.

Vaccines	Type	Cancer	Clinicl outcomes
L-BLP25	Adjuvanted subunit vaccines	NSCLC (phase III)	No difference in overall OS or TTP with placebo. Significantly improved OS in concurrent chemoradiotherapy group.
		NSCLC (phase II)	Significantly improved survival time in stage IIIB NSCLC loco-regional disease compared with supportive care.
		Early BC (phase II)	Neoadjuvant L-BLP25 is safe, but does not improve RCB or pCR rates compared with neoadjuvant standard-of-care treatment.
TG4010	Viral vector vaccines	NSCLC (phase IIb/III)	TG4010 plus chemotherapy improve PFS relative to placebo plus chemotherapy.
		Renal clear-cell carcinoma (phase II)	TG4010 plus cytokines is well tolerated in patients with metastatic renal clear-cell carcinoma.
		Prostate cancer (phase II)	TG4010 is well tolerated, and has biological activity in patients with PSA progression.
STn-KLH	Glycopeptide vaccines	Metastatic BC (phase III)	STn-KLH is well tolerated, but no overall benefit in OS or TTP compared with KLH alone.
Dendritic cell/myeloma fusion vaccine	Dendritic cell vaccines	Multiple myeloma (phase II)	Vaccination following autologous stem cell transplantation can induce significant expansion of myeloma-specific T cells and cytoreduction of minimal residual disease.
Cvac	Dendritic cell vaccines	Epithelial ovarian cancer (phase II)	Cvac immunotherapy is well tolerated and can led to CA125 decline or stabilization.
DC/PANVAC	Dendritic cell vaccines	Colorectal cancer (phase II)	Survival was longer for vaccinated patients than for a contemporary unvaccinated group.
DC/Tn-MUC1	Dendritic cell vaccines	Nonmetastatic castrate-resistant prostate cancer (phase I/II)	Vaccination is safe and can induce significant T-cell responses.
WT1/MUC1-DC	Dendritic cell vaccines	Pancreatic ductal adenocarcinoma (phase I/IIa)	WT1/MUC1-DC vaccination in the adjuvant setting is safe and well-tolerated in pancreatic ductal adenocarcinoma patients after tumor resection.
ImMucin	Adjuvanted subunit vaccines	Multiple myeloma (phase I/II)	ImMucin can induces T- and B-cell specific immune response.
Naked mRNA	RNA vaccines	Renal cell carcinoma (phase I/II)	Vaccination can elicit immunological responses and result in a clinical benefit in patients with metastatic renal cell carcinoma.

OS: overall survival; RCB: residual cancer burden; TTP: time to progression; pCR: pathologic complete response.

## Abbreviations

MUC1: Mucin1
c-Abl: cellular-abelsongene
Ser: serine
Thr: threonine
MUC1-N: N-terminal subunit of MUC1
MUC1-C: C-terminal subunit of MUC1
VNTR: variable number tandem repeat
SEA: sea urchin sperm protein, enterokinase, and agrin
IR: imperfect repeat
CTLs: cytotoxic T lymphocytes
MUC1-CD: cytoplasmic domain of MUC1
ErbB: epidermal growth factor receptor
PKCδ: protein kinase C delta
GSK3: glycogen synthase kinase 3β
EGFR: epidermal growth factor receptor
TF or T: Thomsen-Friedenreich
Tn: Thomsen nouvelle
STn: sialyl-Tn
ST6GAL1: ST6 β-galactoside α-2,6-sialyl -transferase 1
C2GnT-1: core 2 β1,6-N-acetylglucosaminyltransferase-1
RTKs: receptor tyrosine kinases
PI3K: Phosphoinositide 3-kinase
AKT: protein kinase B
PD-L1: programmed cell death-Ligand 1
NF-kB p65: Nuclear factor kB p65
TNBC: triple-negative breast cancer
TAK1: Transforming growth factor-b-activated kinase-1
IKK: IkB kinase
ZEB1: zinc finger E-box binding homolobox 1
VEGF: vascular endothelial growth factor
NRP1: neuropilin-1
MAPK: mitogen-activated protein kinase
JAK: Janus Kinase
STAT: Signal Transducers and Activators of Transcription
mTOR: mammalian target of rapamycin
ERK: extracellular regulated protein kinases
Ras: rat sarcoma
RAF: rapidly accelerated fibrosarcoma
MEK: Mitogen-activated protein kinase kinase
APC: antigen presenting cell
Erα: Estrogen receptors alpha
CCND1: Cyclin D1
TCF4: T-cell factor 4
TCF7L2: Transcription factor 7-like 2
GSK3β: glycogen synthase kinase 3 beta
LEF-1: leukocyte enhancing factor 1
EREs: estrogen-responsive elements
DBD: DNA-binding domain
FLT3: fms-like tyrosine kinase 3
BCR: break-point cluster region
MDSCs: myeloid-derived suppressor cells
AML: acute myeloblastic/myelogenous leukaemia
CML: chronic myelocytic leukemia
ROS: reactive oxygen species
TIGAR: TP53-induced glycolysis and apoptosis regulator
NADPH: Nicotinamide Adenine Dinucleotide Phosphate
GSH: glutathione
MDR: multidrug resistance
MRP1: multidrug resistance-associated protein 1
NSCLC: Non-small cell lung cancers
TLR7: toll-like receptor 7
TLR9: toll-like receptor 9
IFNG: interferon gamma
MCP-1: Monocyte chemoattractant protein-1
GM-CSF: granulocyte-macrophage colony-stimulating factor
ABC: ATP-binding cassette
HIF-1α: hypoxia-inducible factor-1 α
DCs: dendritic cells
TMB: tumor mutational burden
MSI: microsatellite instability
ADCC: antibody-dependent cell-mediated cytotoxicity
ADCP: antibody-dependent cellular phagocytosis
Mab: monoclonal antibody
CARs: Chimeric antigen receptors
CAR T: chimeric antibody receptor T cell
scFv: single chain variable fragment
MHC: major histocompatibility complex
tMUC1: tumor-associated MUC1
PD1: programmed death 1
PSCA: prostate stem cell antigen
NK: natural killer
PBMC: peripheral blood mononuclear cells
KLH: keyhole limpet hemocyanin
BCG: bacillus Calmette-Guerin
MBP: maltose binding protein
QS-21: Quillaja saponaria extract
RCB: pathological complete response
pCR: pathological complete response
IgG: immunoglobulin G
IL-10: interleukin-10
LCP: modified lipid-calcium-phosphate
NP: nanoparticle
Th1: T helper 1
Th2: T helper 2
TAA: tumor-associated antigen
RCC: renal cell carcinoma
IL-2: Interleukin-2
HSP70: heat shock protein70
Ad5: adenovirus 5
PSA: prostate-specific antigen
AuDCs: autologous dendritic cells
M-FP: mannosylated MUC1 protein
nmCRPC: non-metastatic castration resistant prostate cancer
HLA: human leukocyte antigen
PSADT: prostate specific antigen doubling time
TTox: tetanus toxoid
TACA: tumor associated carbohydrate antigen
MMAE: monomethyl whey protein E
ADC: antibody-drug conjugate
MUC1.Tg: MUC1
EGF: transgenic epidermal growth factor
CTLA-4: cytotoxic T-lymphocyte-associated antigen 4
